# Physical Activity and Asthma: A Systematic Review and Meta-Analysis

**DOI:** 10.1371/journal.pone.0050775

**Published:** 2012-12-20

**Authors:** Marianne Eijkemans, Monique Mommers, Jos M. Th. Draaisma, Carel Thijs, Martin H. Prins

**Affiliations:** 1 Department of Pediatrics, Radboud University Medical Centre, Nijmegen, The Netherlands; 2 Department of Epidemiology, School for Public Health and Primary Care (CAPHRI), Maastricht University, Maastricht, The Netherlands; Universidad Peruana de Ciencias Aplicadas (UPC), Peru

## Abstract

**Introduction:**

This review aims to give an overview of available published evidence concerning the association between physical activity and asthma in children, adolescents and adults.

**Methods:**

We included all original articles in which both physical activity and asthma were assessed in case-control, cross-sectional or longitudinal (cohort) studies. Excluded were studies concerning physical fitness, studies in athletes, therapeutic or rehabilitation intervention studies such as physical training or exercise in asthma patients. Methodological quality of the included articles was assessed according to the Newcastle-Ottawa Scale (NOS).

**Results:**

A literature search was performed until June 2011 and resulted in 6,951 publications derived from PubMed and 1,978 publications from EMBASE. In total, 39 studies met the inclusion criteria: 5 longitudinal studies (total number of subjects n = 85,117) with physical activity at baseline as exposure, and asthma incidence as outcome. Thirty-four cross-sectional studies (n = 661,222) were included. Pooling of the longitudinal studies showed that subjects with higher physical activity levels had lower incidence of asthma (odds ratio 0.88 (95% CI: 0.77–1.01)). When restricting pooling to the 4 prospective studies with moderate to good study quality (defined as NOS≥5) the pooled odds ratio only changed slightly (0.87 (95% CI: 0.77–0.99)). In the cross-sectional studies, due to large clinical variability and heterogeneity, further statistical analysis was not possible.

**Conclusions:**

The available evidence indicates that physical activity is a possible protective factor against asthma development. The heterogeneity suggests that possible relevant effects remain hidden in critical age periods, sex differences, or extremes of levels of physical activity (e.g. sedentary). Future longitudinal studies should address these issues.

## Introduction

The prevalence of asthma has increased significantly during the past decades [1]. Concurrently, the prevalence of overweight has increased, while physical activity levels have decreased substantially [2], [3]. In 2005, less than half (49.1%) of US adults met the CDC/ACSM (Centers for Disease Control and Prevention/American College of Sports Medicine) physical activity recommendation (at least 30 minutes of moderately intense activity on five days per week or vigorously intense activity for a minimum of 20 minutes on three days each week) [4]. Physical inactivity is an important risk factor, because it is potentially modifiable and therefore an opportunity for prevention. Several studies have shown that training improves cardiopulmonary fitness, asthma symptoms and quality of life in asthmatic subjects [5]. This evidence suggests that training and high levels of physical activity play a role in the course and severity of asthma. Besides this, an etiological relation between physical activity levels and development of incident asthma might also be possible. Different hypotheses have been suggested to explain the possible protective character of physical activity against asthma development such as reducing airway inflammation, a central feature of asthma [6]. Another explanation is that physical activity could positively influence the patency of bronchioles: poor mucociliary clearance from decreased epithelial stimulation secondary to decreased activity can cause excess mucus and airway edema. Decreased deep inspiration and sigh rate during physical inactivity could lead to smooth muscle latching and subsequent increased risk of asthmatic symptoms [7].

We performed a systematic literature review to evaluate the potential causal relation between physical (in)activity and asthma development, and a pooled analysis to estimate the effect size.

## Methods

### Search strategy

We conducted an electronic search in PubMed (US National Library of Medicine) and EMBASE to obtain all publications on studies that reported on physical activity and asthma published until June 2011. The PubMed search used the Medical Subject Headings (MeSH) terms “motor activity” or text word terms “activity”, “physical activity”, “physical exercise” or “sedentary”, as well as the MeSH term “asthma” or text word terms “asthma”, “asthmatic”, “wheeze” or “wheezing”. The EMBASE search used the MeSH terms “motor activity” or “physical activity” or text word terms “physical activity”, “physical exercise”, “sedentary”, as well as the MeSH terms “asthma” or “wheezing” or the text word terms “asthma”, “asthmatic”, “wheeze” or “wheezing”. These terms were searched using limits that included all articles published in the English language. There were no age restrictions.

We conformed to the MOOSE (Meta-analysis Of Observational Studies in Epidemiology) guidelines for reporting [8] and PRISMA (Preferred Reporting Items for Systematic reviews and Meta-Analyses) statement [9].

### Inclusion

Our primary research question concerned the role of habitual physical activity in the development of incident asthma. Therefore, we searched for longitudinal studies in which the exposure (physical activity) precedes the outcome (onset of asthma). In addition, we included studies that looked into asthma prevalence in different physical activity levels. For this goal, we searched cross-sectional studies that investigated physical activity levels in subjects with asthma compared to controls. For maximal sensitivity, a broad inclusion strategy was used. Inclusion criteria were: original articles in which physical activity as well as asthma was studied, and a control group consisting of healthy subjects or general population. Excluded were studies that did not concern habitual physical activity such as studies in athletes, physical fitness, therapeutic or rehabilitation intervention studies such as physical training in asthma patients. Two investigators (ME, MM) independently assessed whether articles met the inclusion criteria. In case of disagreement, consensus was reached through discussion.

### Quality assessment and data extraction

Methodological quality of included articles was assessed according to the Newcastle-Ottawa Scale (NOS). This instrument was developed to assess the quality of nonrandomized studies. Its content validity and inter-rater reliability has been established [10]. The NOS gives predefined criteria, some of which have to be further specified for the specific topic. We specified these criteria in a consensus meeting with all authors (criteria are presented in figure S1 and S2) before assessing the studies. In short, longitudinal studies were assessed for quality of selection (representativeness, selection of controls, ascertainment of exposure, no asthma at start of study); comparability (confounding); and outcome (assessment of outcome, length and adequacy of follow-up). Gender, weight, and smoking were identified as important confounders. Studies could be awarded a maximum score of 9 points. Studies with scores of 5 points or more were considered to be of moderate to good study quality. However, all studies were used for analysis, irrespective of NOS score. Quality assessment was done by all five authors using the NOS. Each single article was assessed by at least three authors independently. In case of disagreement the other two authors were consulted. Quality assessment was completed before data extraction was started. Data were extracted from the full text article. Quantitative results were extracted from text and tables, choosing preferably those adjusted for important confounders (gender, weight, and smoking). Data-extraction in the longitudinal studies was performed independently by two authors (ME, CT). If essential data were lacking in the original studies, their authors were contacted.

### Statistical analysis

Analyses were performed using the statistical software Review Manager version 5 [11]. Heterogeneity among studies was assessed using the chi-square test (significant at p<0.05) and the Higgins I^2^ test [12]. A random effects model with the Mantel–Haenszel method was used for pooling the results of different studies. Pooled odds ratios (OR) with 95% confidence intervals (CI) were calculated for the longitudinal studies and a subgroup of the cross-sectional studies, namely those studies that used a motion sensor for measuring physical activity levels. We decided to refrain from statistical pooling of the other cross-sectional studies because of substantial clinical and methodological heterogeneity.

## Results

### Literature search

**Figure 1:**
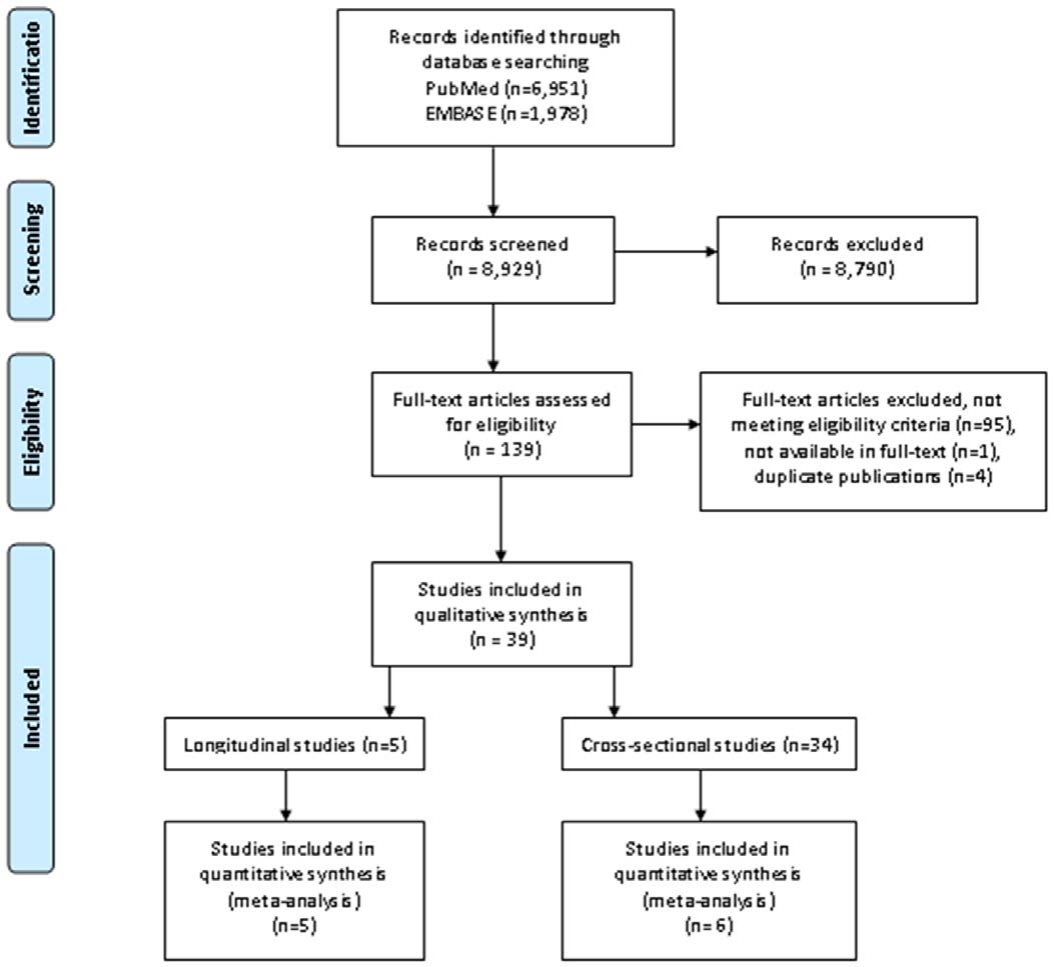
flow diagram of study inclusion.

The search resulted in 6,951 publications derived from PubMed, and 1,978 studies from EMBASE. Based on titles and abstracts, 8,790 articles were excluded at first screening because they did not meet the eligibility criteria, such as experimental studies with intermediate outcomes (such as inflammatory markers) but no asthma as clinical outcome, case reports and studies with case series without control group, and studies of exercise induced asthma in athletes. Full-text copies of the remaining 139 potentially relevant studies were obtained. Ninety-five studies were excluded because they did not meet the inclusion criteria. Four were excluded because they were duplicate publications of the same studies. One study was not available in full-text. The remaining 39 studies were included for this systematic review. Five studies were longitudinal studies and 34 were cross-sectional in design (figure 1).

### Study quality

All 39 studies were assessed using the adjusted NOS scale (see figure S1 and S2 for the adjusted NOS scales), of which the majority (79%) scored 5 points or more, indicating a moderate to good study quality (table S1 and S2). When grouped by study quality, we could not detect a clear pattern in study results or study characteristics (tables 1 and 2). The authors of 4 articles [13], [14], [15], [16] were contacted to obtain essential data that were lacking in the original studies, of which 3 replied [14], [15], [16].

**Table 1:** Overview of longitudinal studies on physical activity at baseline and incident asthma.

Study basics					Physical activity	Asthma	Confounders	Follow up (yrs)	NOS	Odds ratio (95% CI)	Reference
*Reference*	*Country*	*n*	*Age*	*Population*	*Measurement*	*Diagnosis*					
Beckett 2001	US	4,547	18–30	African American and white adults	Questionnaire #c	Asthma medication or doctor's diagnosis (self reported)	Gender, other #h	10	7	2nd quintile: aHR 0.81 (0.57–1.15)3rd quintile: aHR 0.84 (0.59–1.20)4th quintile: aHR 0.94 (0.66–1.35)5th quintile: aHR 1.08 (0.75–1.55)	Highest quintile PA
Benet 2011	France	51,080	40–65	Women	Questionnaire #d	Asthma attacks and doctor's diagnosis (self reported)	Weight, smoking, other #i	9	7	2nd tertile: aHR 1.03 (0.83–1.27)3rd tertile: aHR 1.00 (0.81–1.24)	Lowest tertile PA
Huovinen 2003	Finland	9,671	25–52	Twins#b	Questionnaire #e	Doctor's diagnosis (insurance register)	Gender, weight, smoking, other #j	9	7	Men:Occasional: aOR 0.86 (0.41–1.81)Conditioning: aOR 0.54 (0.22–1.33)Women:Occasional: aOR 1.83 (0.74–4.51)Conditioning: aOR 1.42 (0.51–3.93)	Sedentary
Lucke 2007 #a	Australia	19,021	18–75	Women	Questionnaire #f	Doctor's diagnosis (self reported)	None stated	5–7	6	Older (age 70–75):Nil/low PA: OR 1.15 (0.92–1.47)Mid-aged (age 45–50):Nil/low PA: OR 1.28 (1.09–1.56) *Younger (age 18–23):Nil/low PA: OR 1.12 (0.82–1.54)	Moderate/high PA
Thomsen 2006	Denmark	798	12–41	Discordant twin pairs	Questionnaire #g	Asthma (self reported)	None stated	8	3	Dizygotic twin pairs:High PA: OR 1.48 (0.84–2.61)Monozygotic twin pairs:High PA: OR 0.35 (0.13–0.91) *	Low PA

Overview of study characteristics, study quality based on the Newcastle-Ottawa Scale (NOS) and odds ratios of longitudinal studies on physical activity and asthma incidence. Note that Beckett and Lucke use high physical activity levels as reference category, while Benet, Huovinen and Thomsen use low physical activity levels as reference category.

CI; confidence interval, PA; physical activity, aHR; adjusted hazard ratio, aOR; adjusted odds ratio, OR; odds ratio.

* P<0.05.

#a Data provided by the authors.

#b Analyses did not account for the correlation between twin pairs but twins were considered unrelated subjects.

#c PA assessed through questionnaires using the Physical Activity History Score (validated); categorized in five equal levels (quintiles).

#d PA defined as time spent in household and leisure time PA, converted to metabolic equivalents (METs); categorized in three equal levels (tertiles).

#e Three categories of PA: Sedentary: Respondents estimating their own leisure time physical activity as practically non-existent. Conditioning: Respondents who exercised at least 6 times per month for at least 30 min with a mean intensity corresponding to walking. Occasional: Respondents who did not meet the criteria of sedentary or conditioning.

#f Two categories of PA: Nil/low PA: <600 METs (metabolic equivalents) per week (this reflects 30 minutes of moderate activity on five days each week). Moderate/high PA: >600 METs per week.

#g Two categories of PA: Low PA: <2 hours per week of light leisure time exercise activities. High PA: >2 hours per week of light leisure time exercise activities.

#h other confounders: age, race, centre, and maximal education.

#i other confounders: menopausal status, education level, working status, and co-morbidities.

#j other confounders: age, atopy, and respiratory symptoms.

**Table 2:** Overview of cross-sectional studies on physical activity and asthma prevalence.

Study basics				Physical activity	Asthma	Confounders	NOS	Odds ratio (95% CI)	Reference	Author's conclusions
*Reference*	*Country*	*n*	*Age*	*Measurement*	*Diagnosis*					
**Adults** *Questionnaire*										
Chen 2001	Canada	16,813	>12	Questionnaire #c	Doctor's diagnosis (self reported)	Weight, smoking, other	6			It was concluded that asthmatics were not consistently inactive compared with non-asthmatics.
Dogra 2008	Canada	21,636	65–79	Questionnaire #f	Doctor's diagnosis (self reported)	Gender, weight, other	5			Older asthmatics were less active than their non-asthmatic peers.
Ford 2003	U.S.	165,123	>18	Questionnaire #b d f	Doctor's diagnosis (self reported)	Gender, weight, smoking, other	8			Participants with current asthma were significantly more often considered to be inactive and had significantly lower estimated energy expenditure compared with respondents who never had asthma.
Kilpeläinen 2006	Finland	10,667	18–25	Questionnaire #a	Doctor's diagnosis (self reported)	Gender, weight, smoking, other	7	Men:Moderate PA: aOR 0.62 (0.42–0.92)*Vigorous PA: aOR 0.77 (0.56–1.07)Women:Moderate PA: aOR 0.77 (0.56–1.07)Vigorous PA: aOR 1.19 (0.88–1.60)	low PA	Moderate leisure time physical activity was associated with lower risk of asthma in men, but not among women
Mälkiä 1998	Finland	7,193	>30	Questionnaire #d	Doctor's diagnosis (self reported) and spirometry	Gender	7			The intensity of physical activity was lower in theasthmatic subjects than in those who were not asthmatic.
Ritz 2010	U.S.	40	21–38	Questionnaire #a	Doctor's diagnosis	Other	2			No differences were found between asthma and controls in physical activity.
Strine 2007	U.S.	354,025	>18	Questionnaire #f	Doctor's diagnosis (self reported)	Gender, weight, smoking, other	7	No leisure time PA in past 30 days: aOR 1.2 (1.1–1.2)*	Leisure time PA in past 30 days	Moreover, persons who (…) were physically inactive were slightly more likely to have asthma than those without (…) these behaviors.
Teramoto 2011	U.S.	3,840	>18	Questionnaire #b f	Doctor's diagnosis (self reported)	Gender, weight	6	No regular PA: aOR 3.01 (1.63–5.55)***No leisure time PA in past 30 days: aOR 2.17 (1.40–3.37)***	Regular PA in past 30 days	It was found that asthmatic people spent significantly less time on moderate and vigorous physical activity than their nonasthmatic counterparts.
Vogt 2008	U.S.	4,925	> 18	Questionnaire #b	Doctor's diagnosis (self reported)	Gender, weight, smoking, other	7			[No significant relation between physical activity and asthma diagnosis.]
**Children** *Questionnaire*										
Bener 1996	United Arab Emirates	729	6–14	Questionnaire #f	Asthma symptoms (self reported)	None stated	3			Environmental risk factors associated with asthma were (…) physical exercise. (…)
Cheng 2010	China	232	7–14	Questionnaire #a	Doctor's diagnosis and spirometry	Gender	4			Asthmatic children took part in less exercise than their healthy peers.
Chiang 2006	China	429	9–11	Questionnaire #a b	Doctor's diagnosis	Gender, weight, other	5	MVPA>90 min/week:Healthy controls: OR 1.24 (0.57–2.71)VPA>60 min/week:Healthy controls: OR 2.03 (1.31–3.15)**	Diagnosed asthma	Asthma interferes with children's ability to participate in vigorous physical activity but not in moderate-to-vigorous physical activity.
Corbo 2008	Italy	20,016	6–7	Questionnaire #a e	Asthma symptoms (self reported)	Gender, weight, smoking, other	7	Rarely PA: aOR 1.05 (0.85–1.29)1–2 times/week: aOR 1.13 (0.93–1.38)>3 times/week: aOR 1.33 (0.99–1.77)	No PA	Our data support the hypothesis that (…) spending a lot of time watching television (…) increases the risk of asthma symptoms in children. Wheeze or asthma was not associated with regular sports activity.
Gannotti 2007	U.S.	15,300	9	Questionnaire #a e	Doctor's diagnosis (self reported)	None stated	5			For children with asthma (…), the most frequent perception of parents was that their children were as active as their peers. Days per week of aerobic activity, number of structured activities per week, and playing sports with parents three times a week or more did not vary significantly between children with and without disabilities [including asthma].
Glazebrook 2006	U.K.	117	7–14	Questionnaire #a	Doctor's diagnosis and Peak Flow variability	Gender, weight, other	2			We found that children attending a hospital clinic for asthma (…) were significantly less active than a comparison group with other medical conditions.
Jones 2006	U.S.	13,222	high school (grades 9–12)	Questionnaire #b e f	Doctor's diagnosis (self reported)	Gender, other	6	Sufficient VPA:Current asthma: OR 1.1 (1.0–1.3)Sufficient MPA:Current asthma: OR 1.1 (0.9–1.3)	No asthma	No significant differences were found for participation in sufficient vigorous or moderate physical activity or strengthening exercises among students with and without current asthma.
Kitsantas 2000	U.S.	135	14–18	Questionnaire #a	Doctor's diagnosis	Gender (only girls included)	6			It was found that asthmatic girls (…) participated less often in vigorous activities than nonasthmatic girls.
Lang 2004	U.S.	243	6–12	Questionnaire #a f	Doctor's diagnosis (self reported)	None stated	4			Children with asthma were less active than their peers.
Nystad 1997	Norway	4,585	7–16	Questionnaire #a	Doctor's diagnosis (self reported)	Gender, other	5	PA 1–3 times/week: aOR 0.9 (0.5–1.4)PA>3 times/week: aOR 1.1 (0.6–1.9)	PA<3 times/month	The data suggest that asthmatic children are as physically active as their peers.
Ownby 2007	U.S.	636	8–10	Questionnaire #d	Doctor's diagnosis (self reported)	Gender, other	7			Higher levels of physical activity were related to more diagnosed asthma.
Priftis 2007	Greece	700	10–12	Questionnaire #a d e	Asthma symptoms (self reported)	Gender, weight, other	7	Not participating in any PA: Asthmatic boys: aOR 2.17 (1.34–3.54)* Asthmatic girls: aOR 1.63 (0.86–3.11)	No asthma	Multiple logistic regression analysis revealed that (…) sedentary lifestyle is associated with asthma symptoms only in boys.
Romieu 2004	U.S.	7,851	2–16	Questionnaire #e	Doctor's diagnosis (self reported)	Gender, weight, smoking, other	7	Television watching >4 hours/day: aOR 2.67 (0.97–7.31)	Television watching <3 hours/day	[No significant relation between physical activity and and asthma diagnosis.]
Tsai 2007	China	2,218	11–12	Questionnaire #a e	Doctor's diagnosis (self reported)	Gender, weight, other	6	Boys:PA 1–2 times/week: aOR 0.74 (0.42–1.32)PA>3 times/week: aOR 0.55 (0.30–1.03)PA every day: aOR 0.76 (0.43–1.35)Girls:PA 1–2 times/week: aOR 1.63 (0.69–3.84)PA>3 times/week: aOR 2.27 (0.92–5.59)PA every day: aOR 1.74 (0.67–4.47)	PA low (<1 time/week)	Results of the present study suggest that sedentary life is associated with increased risk of respiratory symptoms. [No significant relation between physical activity and asthma diagnosis.]
Tsai 2009	China	1,287	11–12	Questionnaire #a e	Doctor's diagnosis (self reported)	Gender, weight, smoking, other	7	PA>30 min, times/week: aOR 1.02 (0.96–1.09)	PA <30 min times/week	The number of respiratory symptoms was positively correlated with (…) self-reported sedentary time per weekend-day in girls. [No significant relation between physical activity and asthma diagnosis.]
Vlaski 2008	Macedonia	3,026	13–14	Questionnaire #a e	Doctor's diagnosis (self reported)	Gender, weight, smoking, other	7	VPA 1–2 times/week: aOR 1.84 (0.94–3.60)VPA>3 times/week: aOR 1.13 (0.40–3.23)	VPA occasionally/never	The findings support the aggravating role of sedentary regimen and poor physical fitness on asthma symptoms. [No significant relation between physical activity and asthma diagnosis.]
Vogelberg 2007	Germany	2,910	16–18	Questionnaire #a e	Asthma symptoms (self reported)	Gender, weight, smoking, other	6	Sport>3 times/week: aOR 0.8 (0.5–1.3)	Sport <1 time/month	In the bivariate analyses, exercising more than once per week (…) was inversely related to new onset of wheeze. The association between physical activity and new onset of wheeze disappeared when active smoking was taken into account.
Weston 1989	New Zealand	408	11–13	Questionnaire #a	Doctor's diagnosis (self reported)	None stated	4			Asthmatic children were significantly more active than nonasthmatic children for all activities and for school activities.
**Children** *Motion sensor*										
Berntsen 2009	Norway	174	13–14	Accelero-meter SenseWear #a c	Doctor's diagnosis (self reported)	Gender, other	7			Neither aerobic fitness, total energy expenditure nor hours in moderate to very vigorous intensity physical activity during week and weekend differed between adolescents with and without asthma.
Eijkemans 2008	The Netherlands	305	4–5	Accelero-meter Actigraph and Questionnaire #a	Asthma symptoms (self reported)	Smoking, other	6	Total activity (counts/minute)Boys:Recent wheeze: aGMR 1.06 (0.94–1.20) Girls:Recent wheeze: aGMR 0.99 (0.85–1.14)	Never wheeze	Our data provide no evidence that asthmatic symptoms induce a lower physical activity level.
Firrincieli 2005	U.S.	54	3–5	Accelero-meter Actiwatch #a	Asthma symptoms (self reported)	None stated	5			Physical activity measured with the motion sensor was decreased among children with a history of wheezing.
Rundle 2009	U.S.	437	4	Accelero-meter Actiwatch #a e	Doctor's diagnosis or wheeze (both self reported)	Weight, other	5	Quartile of mean activity counts/minuteQuartile 2: aOR 0.85 (0.45–1.63)Quartile 3: aOR 1.03 (0.54–1.96)Quartile 4: aOR 0.91 (0.46–1.80)	Quartile 1 (lowest PA)	In cross-sectional analyses (…) asthma symptoms were not associated with physical activity in this age group.
Vahlkvist 2009	Denmark	214	6–14	Accelero-meter RT3 #a d	Asthma symptoms (self reported) and FEV variability	None stated	4			No statistically significant differences were found between the two groups [asthma vs no asthma] in overall daily activity, time spent in high or vigorous activity (…)
Van Gent 2007	The Netherlands	1,614	7–10	Accelero-meter PAM and Questionnaire #a	Doctor's diagnosis (self reported) and FEV variability	None stated	6			Childhood asthma does not appear to be associated with a decreased level of daily physical activity in our study population.
Walders-Abramson 2009	U.S.	118	10–16	Pedometer Omron #a	Doctor's diagnosis (self reported) and asthma medication	None stated	7			We found similar rates of objectively measured physical activity among youth with well controlled asthma and controls.

Overview of study characteristics, study quality based on the Newcastle-Ottawa Scale (NOS), odds ratios and author's conclusions of cross-sectional studies on physical activity and asthma prevalence. Odds ratios are noted here only if odds ratios or equivalents with 95% confidence intervals are specified in the article. Author's conclusions are noted only if the author mentions a conclusion on the relation between physical activity and asthma prevalence. If not, a conclusion was drawn based on the data in the article. In this case the conclusion is noted between [ ].

CI; confidence interval, PA; physical activity, aOR; adjusted odds ratio, aHR; adjusted hazard ratio, OR; odds ratio, aGMR; adjusted geometric mean ratio, MVPA; moderate to vigorous physical activity, VPA; vigorous physical activity.

* P<0.05.

** P<0.01.

*** P<0.001.

#a frequency of physical activity (PA).

#b participation of enough PA to meet the recommendations for PA.

#c Energy Expenditure (EE).

#d Metabolic Equivalent of Task (MET).

#e physical inactivity (e.g. TV watching, computer play).

#f physically active vs. physically not active group.

### Longitudinal studies

Study characteristics and odds ratios of the longitudinal studies [14], [17], [18], [19], [20] are summarized in table 1. All 5 studies looked into physical activity levels of subjects at baseline and incident asthma during follow up. Follow up duration ranged between 5 and 10 years. Physical activity was assessed by questionnaires. Different reference categories, subgroups and confounders were used (table 1). Asthma diagnosis was defined as doctor's diagnosis, either through self report or linkage to an insurance registry.

### Statistical analysis and pooling

**Figure 2:**
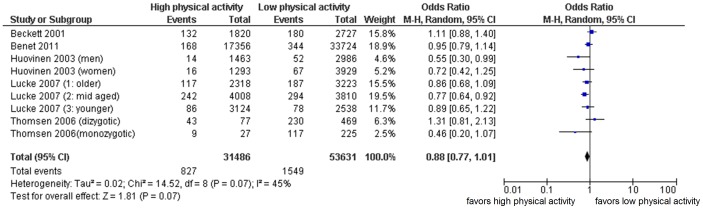
pooling of longitudinal data: physical activity at baseline and risk of asthma incidence. M-H; Mantel-Haenszel method, Random effects, CI; confidence interval. Not adjusted for potential confounders. Low physical activity used as reference category. Note that odds ratios are different of those in table 1 because reference categories were reversed and/or the number of categories was converted into two categories per study. For example Beckett et al. and Lucke et al. use high physical activity as reference category; in our meta-analysis we standardized low physical activity as reference category. In studies were more than two categories of physical activity were used (such as Beckett et al. who used 5 levels of physical activity), these were converted into two categories (in case of Becket et al. we converted the highest two levels into high physical activity, and the lowest three levels into low physical activity).

Data of the 5 longitudinal studies were pooled using a random effects model (figure 2). Data on studies with more than two groups of different physical activity levels were converted into two groups, namely low physical activity and high physical activity, of which low physical activity was used as reference category. In case of an uneven number of groups, the reference category consisted of the lowest physical activity levels including the middle group.

Pooled odds ratio was 0.88 (95% CI: 0.77–1.01). Chi-square test for heterogeneity was borderline significant (p = 0.07). Higgins I^2^ index was 45%, indicating moderate inconsistency. These results are not adjusted for potential confounders as the majority of studies did not provide adjusted results. When we restricted analysis to the studies with moderate to good study quality, identified by NOS scores of 5 or higher, 4 studies remained [14], [17], [18], [19]. Sensitivity analysis showed a consistent result: the pooled odds ratio did not change much (0.87 (95% CI: 0.77–0.99)) but did reach statistical significance.

### Cross-sectional studies

Study characteristics and results of the 34 cross-sectional studies [13], [15], [16], [21], [22], [23], [24], [25], [26], [27], [28], [29], [30], [31], [32], [33], [34], [35], [36], [37], [38], [ 39], [40], [41], [42], [43], [44], [45], [46], [47], [48], [49], [50], [51], [52] are summarized in table 2. The vast majority (25 studies) examined children of different age spans, 8 studies included only adults, and one study included both children (12 years and older) and adults (table 2).

**Figure 3:**
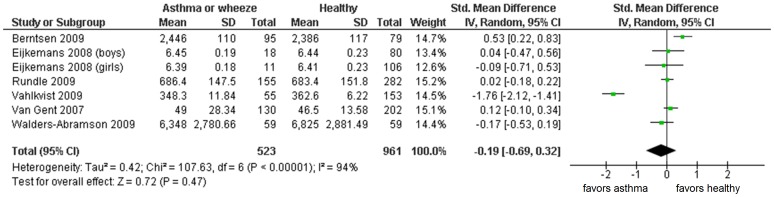
pooling of cross-sectional data using motion sensors: physical activity measured by motion sensors and asthma prevalence. Random effects, CI; confidence interval. Not adjusted for potential confounders. Low physical activity used as reference category.

Physical activity was assessed by motion sensors in 7 studies [13], [16], [22], [28], [42], [48], [49], all examining children (n = 2916). In these studies, asthma was defined as self reported doctor's diagnosis or asthma symptoms. Two studies [48], [49] combined self reported asthma diagnosis with spirometry. Not enough data were available for pooling in one study (i.e. standard deviations were missing despite contacting the authors). [13] Data of the other 6 studies using motion sensors were pooled: standard mean difference −0.19 (95% CI −0.69; +0.32) (figure 3). Testing for heterogeneity showed a highly significant result (p<0.00001) and high inconsistency (I^2^ = 94%).

The other 27 studies used only questionnaires to assess physical activity levels. Methods were diverse (table 2): the majority focused on activity by counting frequency and duration of activity per time unit (month, week, day). [15], [24], [25], [26], [30], [31], [33], [34], [35], [37], [39], [40], [46], [47], [50], [52] Others looked into the proportion of subjects that was physically active [21], [27], [29], [32], [35], [43], [44] or met the physical activity recommendation. [25], [29], [32], [44], [51] A relatively small number of studies focused on energy expenditure per day [23] or metabolic equivalent of task (MET). [29], [36], [38], [39] Besides physical activity, inactivity (television watching, sedentary time) was also investigated by 9 studies. [15], [26], [30], [32], [39], [41], [46], [47], [50] Asthma was defined as self reported doctor's diagnosis or asthma symptoms. Only three studies combined questionnaire based asthma diagnosis with spirometry. [24], [31], [36]

We decided to refrain from statistical pooling due to heterogeneity of study designs, populations, and measurement methods for both physical activity and asthma outcome.

In total, 13 studies (564,394 subjects in total) reported a statistically significant association between high physical activity levels and lower asthma prevalence. [13], [24], [25], [27], [29], [31], [33], [34], [35], [36], [39], [43], [44] In contrast, 3 studies (total of 1,773 subjects) found a statistically significant association between high physical activity levels and higher asthma prevalence. [21], [38], [52] Eighteen studies (95,055 subjects) obtained no significant results. [15], [16], [22], [23], [26], [28], [30], [32], [37], [40], [41], [42], [46], [47], [48], [49], [50], [51]

## Discussion

This systematic review gives an overview of the published evidence concerning the association between physical activity and asthma. Our primary research question was aimed at the etiological association between different physical activity levels and subsequent asthma incidence. In an extensive search, we only found 5 longitudinal studies that met the inclusion criteria and could be of use in answering this question. Although the number of longitudinal studies was small, the total accrued number of subjects was considerable (n = 85,117). Pooling showed that subjects with higher physical activity levels might have lower risk of developing asthma.

Thirty-four studies were cross-sectional in design. Due to large clinical variability and heterogeneity we had to refrain from further statistical analysis, except for a small group of studies using a motion sensor to measure physical activity. Despite this limitation, however, we can draw some conclusions: a substantial number of included cross-sectional studies, with the largest total study population, did find an association between high physical activity levels and low asthma prevalence. This seems consistent with physical activity being protective against asthma. However, we can not rule out publication bias. Moreover, cross-sectional studies are not suited to give insight into the causal relation between physical activity and subsequent asthma incidence. Besides the hypothesis that subjects with higher physical activity levels have a lower risk of developing asthma (protective), reverse causality is also possible. There are several hypotheses why asthma patients (with asthma as exposure) could have lower physical activity levels (outcome), such as fear for symptoms of shortness of breath, wrongful education, or by asthma that is not well regulated.

In contrast to the studies that were cross-sectional in design, this reverse causality does not play a role in interpreting the results of the 5 longitudinal studies. In all 5 studies physical activity levels were measured before asthma was diagnosed. However, the results could be influenced by protopathic bias (e.g. physical activity restricted by respiratory complaints that precede an asthma diagnosis) or earlier diagnosis of asthma through exercise-induced symptoms. The first would lead to overestimation of the true association between low physical activity levels and subsequent asthma development; whereas the second would lead to an underestimation. Unfortunately, none of the longitudinal studies addressed these biases.

### Limitations

It is important to realize that there are several limitations to this review. First of all, due to the fact that this research is based on published material, publication bias is an important factor. Furthermore, studies showed substantial heterogeneity in different areas such as population (number, age, gender, race, duration of follow-up), exposure variables (physical activity measured by questionnaires, whether or not validated, or measured by motion sensors) and outcome variables (asthma diagnosis as self reported doctor's diagnosis, asthma symptoms or spirometry). Analysis showed borderline statistical heterogeneity. The small number of longitudinal studies prevented us from performing meta-regression or subgroup analysis. Confounding is an important issue, because other risk factors (such as smoking and obesity) could be associated with both low habitual physical activity as well as asthma development. First and second-hand cigarette smoke exposure is already established as an independent risk factor for developing asthma. [53] It is suggested that obesity is a risk factor for asthma development. [54] In our meta-analysis of longitudinal studies, pooling of results adjusted for confounders was not sensible because only three studies presented such results. However, adjusted odds ratios were never lower than unadjusted odds ratios (see table S3), so that a pooled effect for the adjusted results would be higher than the odds ratio of 0.88 (95% CI: 0.77–1.01) found for the unadjusted result.

Another limitation might be the fact that the validity of the NOS score recently has been questioned by Stang who believes that the NOS provides a quality score that has unknown validity at best. [55] We noted that some methodological pitfalls were not well represented in the NOS scale: reverse causation and protopathic bias or confounding by indication (e.g. advice to remain physically active for children with respiratory complaints).

### Conclusion

In conclusion, the results of available published evidence indicate that high physical activity levels are a possible protective factor against asthma development. The heterogeneity suggests that possible relevant effects remain hidden in critical age periods, sex differences, or extremes of levels of physical activity (e.g. sedentary). Future longitudinal studies should address these issues.

## Supporting Information

Figure S1 **NOS scale physical activity and asthma longitudinal studies.** NOS: Newcastle-Ottawa Scale. Adjusted NOS scale for physical activity and asthma in longitudinal studies.
(DOC)

Figure S2 **NOS scale physical activity and asthma cross-sectional studies.** NOS: Newcastle-Ottawa Scale. Adjusted NOS scale for physical activity and asthma in cross-sectional studies.
(DOC)

Figure S3 **PRISMA 2009 Checklist.** PRISMA (Preferred Reporting Items for Systematic reviews and Meta-Analyses) checklist adjusted for this study.
(DOC)

Table S1 **NOS scores of longitudinal studies.** NOS: Newcastle-Ottawa Scale. Result of quality assessment of longitudinal studies on physical activity and asthma using NOS scores. We refer to figure S1 for the adjusted NOS for longitudinal studies, which was used as a scoring list.
(DOC)

Table S2 **NOS scores of cross-sectional studies.** NOS: Newcastle-Ottawa Scale. Result of quality assessment of cross-sectional studies on physical activity and asthma using NOS scores. We refer to figure S2 for the adjusted NOS for cross-sectional studies, which was used as a scoring list.
(DOC)

Table S3 **Data extraction of longitudinal studies.** CI; confidence interval, PA; physical activity, aHR; adjusted hazard ratio, OR; odds ratio; aOR; adjusted odds ratio, BMI; body mass index. Data extraction of longitudinal studies concerning baseline physical activity and asthma incidence. * p<0.05, #a used as reference category for pooling in this review, #1 adjusted for age, race, sex, center, and maximal education, #2 adjusted for BMI, smoking status, menopausal status, education level, working status, co-morbidities, #3 adjusted for age, atopy, and respiratory symptoms.
(DOC)
