# Supplementary material for: Physical Activity and Asthma: A Systematic Review and Meta-Analysis
Source: PLoS One. 2012 Dec 20;7(12):e50775. doi: 10.1371/journal.pone.0050775 (PMC3527462; doi:10.1371/journal.pone.0050775)
Supplement: Figure S1 — NOS scale physical activity and asthma longitudinal studies. NOS: Newcastle-Ottawa Scale. Adjusted NOS scale for physical activity and asthma in longitudinal studies. (DOC) [file pone.0050775.s001.doc]

**NEWCASTLE - OTTAWA QUALITY ASSESSMENT SCALE**

**COHORT STUDIES**

**Review: physical activity and asthma**

Note: A study can be awarded a maximum of one star for each numbered item within the Selection and Outcome categories. A maximum of two stars can be given for Comparability

**Selection**

1) Representativeness of the exposed cohort

a) truly representative of the general population **🟑**

b) somewhat representative of the general population **🟑**

c) selected group of users eg nurses, volunteers

d) no description of the derivation of the cohort

2) Selection of the non exposed cohort

a) drawn from the same community as the exposed cohort **🟑**

b) drawn from a different source

c) no description of the derivation of the non exposed cohort

3) Ascertainment of exposure

a) use of accelerometer **🟑**

b) validated questionnaire **🟑**

c) not validated questionnaire or no validation is mentioned

d) no description

4) Demonstration that outcome of interest was not present at start of study (no asthma at start of study)

a) yes **🟑**

b) no

**Comparability**

1) Comparability of cohorts on the basis of the design or analysis

a) study controls for gender **🟑**

b) study controls for any smoking (eg. parental smoking, past smoking, smoking during pregnancy) AND weight (eg. BMI, overweight, obesity) **🟑**

**Outcome**

1) Assessment of outcome

a) doctor’s diagnosis (not self reported doctor’s diagnosis) OR objective measurements (eg. lungfunction) **🟑**

b) parent/self reported doctor’s diagnosis OR use of asthma medication **🟑**

c) parent/self report

d) no description

2) Was follow-up long enough for outcomes to occur

a) yes (majority of population at least 1 year) **🟑**

b) no

3) Adequacy of follow up of cohorts

a) complete follow up - all subjects accounted for **🟑**

b) subjects lost to follow up unlikely to introduce bias - small number lost - > 80 % follow up, or description provided of those lost, proving a non-selective loss to follow up **🟑**

c) follow up rate < 80% and no description of those lost, or a selective loss to follow up

d) no statement
