# Supplementary material for: Physical Activity and Asthma: A Systematic Review and Meta-Analysis
Source: PLoS One. 2012 Dec 20;7(12):e50775. doi: 10.1371/journal.pone.0050775 (PMC3527462; doi:10.1371/journal.pone.0050775)
Supplement: Figure S2 — NOS scale physical activity and asthma cross-sectional studies. NOS: Newcastle-Ottawa Scale. Adjusted NOS scale for physical activity and asthma in cross-sectional studies. (DOC) [file pone.0050775.s002.doc]

**NEWCASTLE - OTTAWA QUALITY ASSESSMENT SCALE**

**CASE CONTROL STUDIES**

**Review: physical activity and asthma**

Note: A study can be awarded a maximum of one star for each numbered item within the Selection and Exposure categories. A maximum of two stars can be given for Comparability.

**Selection**

1) Is the case definition (asthma) adequate?

a) yes, with independent validation (eg. self reported doctor’s diagnosis, reference tot primary record source) **🟑**

b) yes, based on self reports

c) no description

2) Representativeness of the cases

a) consecutive or obviously representative series of cases (random sample of cases) **🟑**

b) potential for selection biases or not stated

3) Selection of Controls

a) community controls (same community as cases)**🟑**

b) hospital controls

c) no description

4) Definition of Controls

a) no history of disease (endpoint) **🟑**

b) no description of source

**Comparability**

1) Comparability of cases and controls on the basis of the design or analysis

a) study controls for gender **🟑**

b) study controls for any smoking (eg. parental smoking, past smoking, smoking during pregnancy) AND weight (eg. BMI, overweight, obesity) **🟑**

**Exposure**

1) Ascertainment of exposure

a) use of accelerometer **🟑**

b) validated questionnaire **🟑**

c) not validated questionnaire or no validation is mentioned

d) written self report

e) no description

2) Same method of ascertainment for cases and controls

a) yes **🟑**

b) no

3) Non-Response rate

a) same rate for both groups **🟑**

b) non respondents described

c) rate different and no designation
