# Supplementary material for: Physical Activity and Asthma: A Systematic Review and Meta-Analysis
Source: PLoS One. 2012 Dec 20;7(12):e50775. doi: 10.1371/journal.pone.0050775 (PMC3527462; doi:10.1371/journal.pone.0050775)
Supplement: Table S1 — NOS scores of longitudinal studies. NOS: Newcastle-Ottawa Scale. Result of quality assessment of longitudinal studies on physical activity and asthma using NOS scores. We refer to figure S1 for the adjusted NOS for longitudinal studies, which was used as a scoring list. (DOC) [file pone.0050775.s004.doc]

| **NOS scale** | **Beckett 2001** | **Benet 2011** | **Huovinen 2002** | **Lucke 2007** | **Thomsen 2006** |
| --- | --- | --- | --- | --- | --- |
| **A Selection (maximum 4)** | **4** | **3** | **3** | **3** | **2** |
| 1 Representativeness of the exposed cohort | 1 | 0 | 1 | 1 | 0 |
| 2 Selection of the non exposed cohort | 1 | 1 | 1 | 1 | 1 |
| 3 Ascertainment of exposure | 1 | 1 | 0 | 0 | 0 |
| 4 Demonstration that outcome of interest was not present at start of study | 1 | 1 | 1 | 1 | 1 |
|  |  |  |  |  |  |
| **B Comparability (maximum 2)** | **1** | **2** | **2** | **1** | **0** |
| 1 Comparability of cohorts on the basis of the design of analysis | 1 | 2 | 2 | 1 | 0 |
|  |  |  |  |  |  |
| **C Outcome (maximum 3)** | **2** | **2** | **2** | **2** | **1** |
| 1 Assessment of outcome | 1 | 1 | 1 | 1 | 0 |
| 2 Was follow-up long enough for outcomes to occur | 1 | 1 | 1 | 1 | 1 |
| 3 Adequacy of follow up of cohorts | 0 | 0 | 0 | 0 | 0 |
|  |  |  |  |  |  |
| **Totaal (maximum 9)** | **7** | **7** | **7** | **6** | **3** |

Table S1: NOS scores of longitudinal studies.
