# Supplementary material for: Physical Activity and Asthma: A Systematic Review and Meta-Analysis
Source: PLoS One. 2012 Dec 20;7(12):e50775. doi: 10.1371/journal.pone.0050775 (PMC3527462; doi:10.1371/journal.pone.0050775)
Supplement: Table S2 — NOS scores of cross-sectional studies. NOS: Newcastle-Ottawa Scale. Result of quality assessment of cross-sectional studies on physical activity and asthma using NOS scores. We refer to figure S2 for the adjusted NOS for cross-sectional studies, which was used as a scoring list. (DOC) [file pone.0050775.s005.doc]

| **NOS scale** | **Bener 1996** | **Berntsen 2009** | **Chen 2001** | **Cheng 2010** | **Chiang 2006** | **Corbo 2008** | **Dogra 2008** | **Eijkemans 2008** | **Firrincieli 2005** | **Ford 2003** | **Gannotti 2007** | **Glazebrook 2006** | **Jones 2006** | **Kilpeläinen 2006** | **Kitsantas 2000** | **Lang 2004** | **Mälkiä 1998** |
| --- | --- | --- | --- | --- | --- | --- | --- | --- | --- | --- | --- | --- | --- | --- | --- | --- | --- |
| **A Selection (maximum 4)** | **2** | **4** | **3** | **2** | **2** | **4** | **3** | **3** | **3** | **4** | **4** | **1** | **4** | **4** | **4** | **2** | **4** |
| 1 Case definition adequate | 0 | 1 | 1 | 1 | 1 | 1 | 1 | 0 | 0 | 1 | 1 | 1 | 1 | 1 | 1 | 1 | 1 |
| 2 Representativeness of the cases | 1 | 1 | 1 | 0 | 0 | 1 | 1 | 1 | 1 | 1 | 1 | 0 | 1 | 1 | 1 | 0 | 1 |
| 3 Selection of controls | 1 | 1 | 1 | 0 | 0 | 1 | 1 | 1 | 1 | 1 | 1 | 0 | 1 | 1 | 1 | 0 | 1 |
| 4 Definition of controls | 0 | 1 | 0 | 1 | 1 | 1 | 0 | 1 | 1 | 1 | 1 | 0 | 1 | 1 | 1 | 1 | 1 |
|  |  |  |  |  |  |  |  |  |  |  |  |  |  |  |  |  |  |
| **B Comparability (maximum 2)** | **0** | **1** | **2** | **1** | **1** | **2** | **1** | **1** | **0** | **2** | **0** | **0** | **1** | **2** | **1** | **0** | **1** |
| 1 Comparability of cohorts on the basis of the design of analysis | 0 | 1 | 2 | 1 | 1 | 2 | 1 | 1 | 0 | 2 | 0 | 0 | 1 | 2 | 1 | 0 | 1 |
|  |  |  |  |  |  |  |  |  |  |  |  |  |  |  |  |  |  |
| **C Exposure (maximum 3)** | **1** | **2** | **1** | **1** | **2** | **1** | **1** | **2** | **2** | **2** | **1** | **1** | **1** | **1** | **1** | **2** | **2** |
| 1 Ascertainment of exposure | 0 | 1 | 0 | 0 | 1 | 0 | 0 | 1 | 1 | 1 | 0 | 0 | 0 | 0 | 0 | 1 | 1 |
| 2 same method cases and controls? | 1 | 1 | 1 | 1 | 1 | 1 | 1 | 1 | 1 | 1 | 1 | 1 | 1 | 1 | 1 | 1 | 1 |
| 3 non-response rate | 0 | 0 | 0 | 0 | 0 | 0 | 0 | 0 | 0 | 0 | 0 | 0 | 0 | 0 | 0 | 0 | 0 |
|  |  |  |  |  |  |  |  |  |  |  |  |  |  |  |  |  |  |
| **Totaal (maximum 9)** | **3** | **7** | **6** | **4** | **5** | **7** | **5** | **6** | **5** | **8** | **5** | **2** | **6** | **7** | **6** | **4** | **7** |

Table S2: NOS scores of cross-sectional studies.

| **NOS scale** | **Nystad 1997** | **Ownby 2007** | **Priftis 2007** | **Ritz 2010** | **Romieu 2004** | **Rundle 2009** | **Strine 2007** | **Teramoto 2011** | **Tsai 2007** | **Tsai 2009** | **Vahlkvist 2009** | **Van Gent 2007** | **Vlaski 2008** | **Vogelberg 2007** | **Vogt 2008** | **Walders-Abramson 2009** | **Weston 1989** |
| --- | --- | --- | --- | --- | --- | --- | --- | --- | --- | --- | --- | --- | --- | --- | --- | --- | --- |
| **A Selection (maximum 4)** | **3** | **4** | **4** | **1** | **4** | **3** | **4** | **4** | **4** | **4** | **2** | **4** | **4** | **3** | **4** | **3** | **3** |
| 1 Case definition adequate | 0 | 1 | 1 | 1 | 1 | 0 | 1 | 1 | 1 | 1 | 1 | 1 | 1 | 0 | 1 | 1 | 0 |
| 2 Representativeness of the cases | 1 | 1 | 1 | 0 | 1 | 1 | 1 | 1 | 1 | 1 | 0 | 1 | 1 | 1 | 1 | 0 | 1 |
| 3 Selection of controls | 1 | 1 | 1 | 0 | 1 | 1 | 1 | 1 | 1 | 1 | 0 | 1 | 1 | 1 | 1 | 1 | 1 |
| 4 Definition of controls | 1 | 1 | 1 | 0 | 1 | 1 | 1 | 1 | 1 | 1 | 1 | 1 | 1 | 1 | 1 | 1 | 1 |
|  |  |  |  |  |  |  |  |  |  |  |  |  |  |  |  |  |  |
| **B Comparability (maximum 2)** | **1** | **1** | **1** | **0** | **2** | **0** | **2** | **1** | **1** | **2** | **0** | **0** | **2** | **2** | **2** | **1** | **0** |
| 1 Comparability of cohorts on the basis of the design of analysis | 1 | 1 | 1 | 0 | 2 | 0 | 2 | 1 | 1 | 2 | 0 | 0 | 2 | 2 | 2 | 1 | 0 |
|  |  |  |  |  |  |  |  |  |  |  |  |  |  |  |  |  |  |
| **C Exposure (maximum 3)** | **1** | **2** | **2** | **1** | **1** | **2** | **1** | **1** | **1** | **1** | **2** | **2** | **1** | **1** | **1** | **3** | **1** |
| 1 Ascertainment of exposure | 0 | 1 | 1 | 0 | 0 | 1 | 0 | 0 | 0 | 0 | 1 | 1 | 0 | 0 | 0 | 1 | 0 |
| 2 same method cases and controls? | 1 | 1 | 1 | 1 | 1 | 1 | 1 | 1 | 1 | 1 | 1 | 1 | 1 | 1 | 1 | 1 | 1 |
| 3 non-response rate | 0 | 0 | 0 | 0 | 0 | 0 | 0 | 0 | 0 | 0 | 0 | 0 | 0 | 0 | 0 | 1 | 0 |
|  |  |  |  |  |  |  |  |  |  |  |  |  |  |  |  |  |  |
| **Totaal (maximum 9)** | **5** | **7** | **7** | **2** | **7** | **5** | **7** | **6** | **6** | **7** | **4** | **6** | **7** | **6** | **7** | **7** | **4** |
