# Supplementary material for: Physical Activity and Asthma: A Systematic Review and Meta-Analysis
Source: PLoS One. 2012 Dec 20;7(12):e50775. doi: 10.1371/journal.pone.0050775 (PMC3527462; doi:10.1371/journal.pone.0050775)
Supplement: Table S3 — Data extraction of longitudinal studies. CI; confidence interval, PA; physical activity, aHR; adjusted hazard ratio, OR; odds ratio; aOR; adjusted odds ratio, BMI; body mass index. Data extraction of longitudinal studies concerning baseline physical activity and asthma incidence. * p<0.05, #a used as reference category for pooling in this review, #1 adjusted for age, race, sex, center, and maximal education, #2 adjusted for BMI, smoking status, menopausal status, education level, working status, co-morbidities, #3 adjusted for age, atopy, and respiratory symptoms. (DOC) [file pone.0050775.s006.doc]

| **Study** | **Unadjusted results** | | | **Adjusted results** | |
| --- | --- | --- | --- | --- | --- |
|  |  |  |  | **(95% CI)** |  |
| **Beckett 2001** |  | Not reported |  | **aHR** #1 |  |
| *1st quintile (highest PA)* |  |  |  | Reference |  |
| *2nd quintile* |  |  |  | 0.81 (0.57-1.15) |  |
| *3rd quintile #a* |  |  |  | 0.84 (0.59-1.20) |  |
| *4th quintile #a* |  |  |  | 0.94 (0.66-1.35) |  |
| *5th quintile (lowest PA) #a* |  |  |  | 1.08 (0.75-1.55) |  |
|  |  |  |  |  |  |
| **Benet 2011** |  | Not reported |  | **aHR** #2 |  |
| *1st tertile (lowest PA) #a* |  |  |  | Reference |  |
| *2nd tertile #a* |  |  |  | 1.03 (0.83-1.27) |  |
| *3rd tertile (highest PA)* |  |  |  | 1.00 (0.81-1.24) |  |
|  |  |  |  |  |  |
| **Huovinen 2003** | **OR** |  |  | **aOR** #3 |  |
|  | *men* | *women* |  | *men* | *women* |
| *Sedentary #a* | Reference | Reference |  | Reference | Reference |
| *Occasional #a* | 0.82 (0.40-1.68) | 1.71 (0.69-4.21) |  | 0.86 (0.41-1.81) | 1.83 (0.74-4.51) |
| *Conditioning* | 0.47 (0.20-1.10) | 1.22 (0.44-3.34) |  | 0.54 (0.22-1.33) | 1.42 (0.51-3.93) |
|  |  |  |  |  |  |
| **Lucke 2007** | **RR** |  |  |  |  |
|  | *Younger cohort (age 18-23)* | *Mid-aged cohort (age 45-50)* | *Older cohort (age 70-75)* | Not reported |  |
| *Moderate/high PA* | Reference | Reference | Reference |  |  |
| *Nil/low PA #a* | 1.12 (0.82-1.54) | 1.28 (1.09-1.56)* | 1.15 (0.92-1.47) |  |  |
|  |  |  |  |  |  |
|  | **OR** |  |  |  |  |
| **Thomsen 2006** | *Monozygotic twin pairs* | *Dizygotic twin pairs* |  | Not reported |  |
| *Low PA #a* | Reference | Reference |  |  |  |
| *High PA* | 0.35 (0.13-0.91)* | 1.48 (0.84-2.61) |  |  |  |

Table S3: data extraction of longitudinal data.
